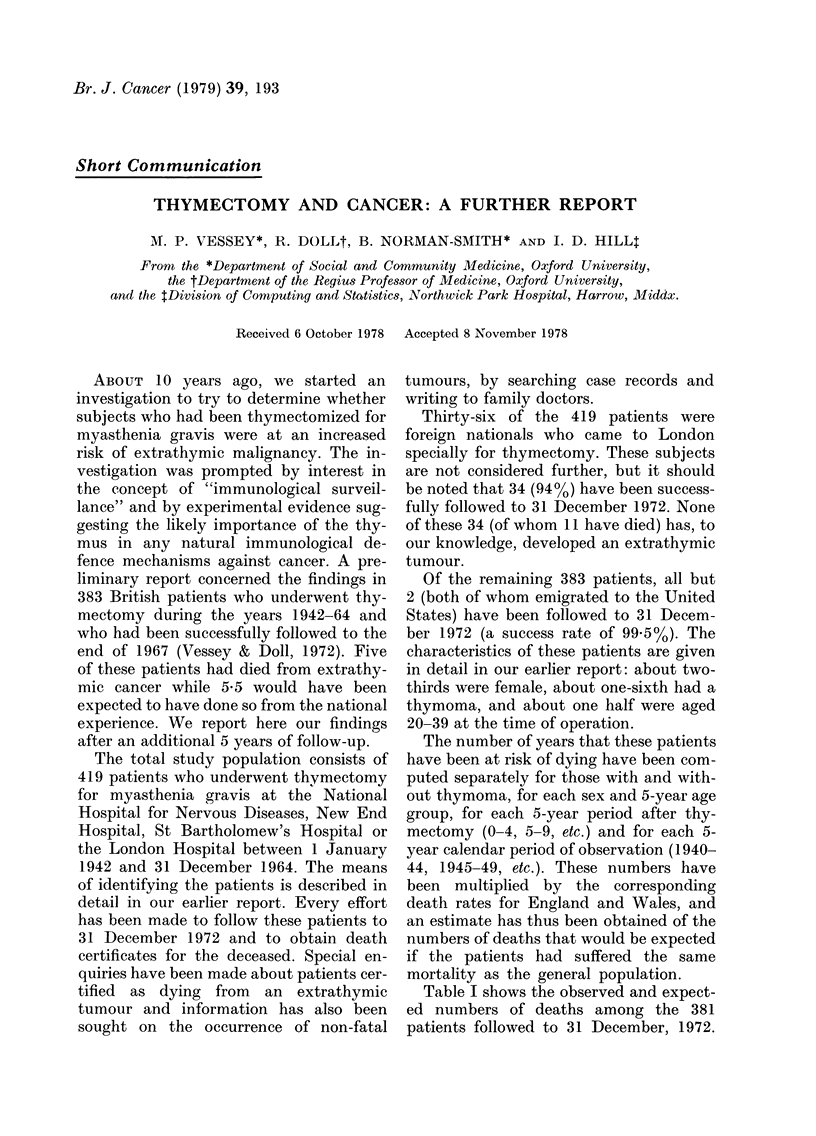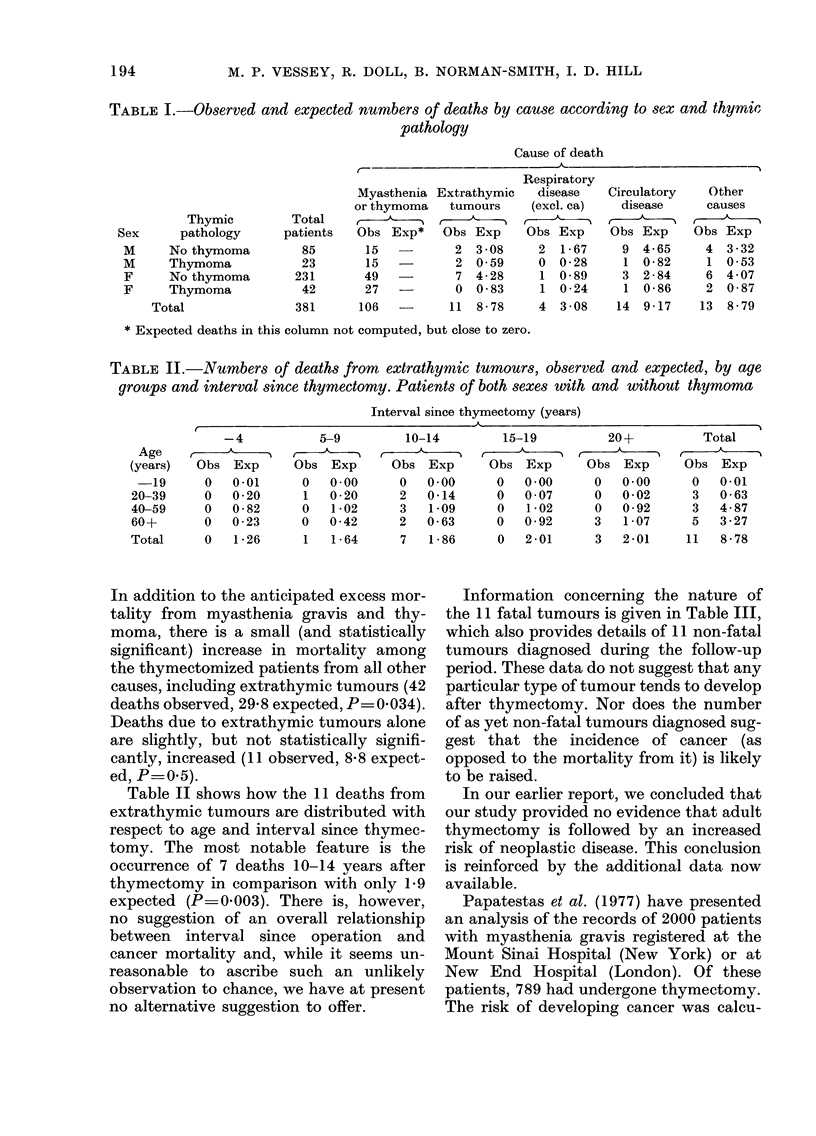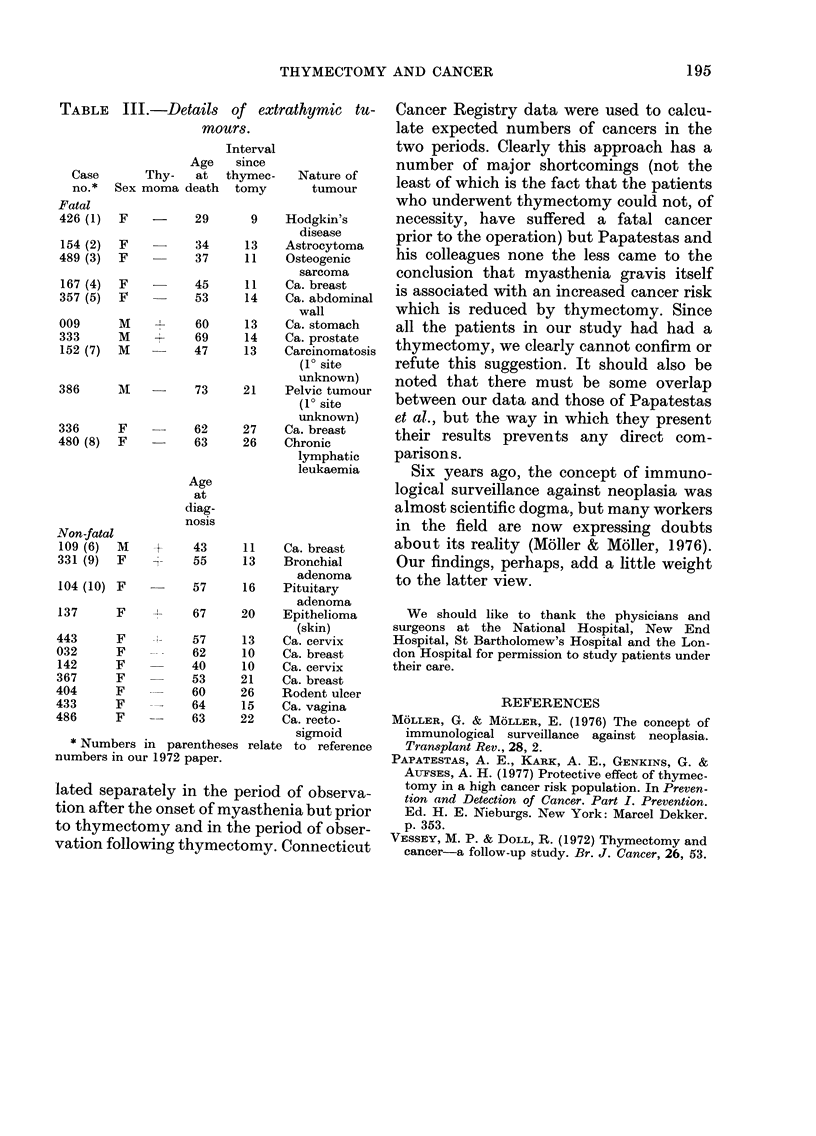# Thymectomy and cancer: a further report.

**DOI:** 10.1038/bjc.1979.30

**Published:** 1979-02

**Authors:** M. P. Vessey, R. Doll, B. Norman-Smith, I. D. Hill


Br. J. Cancer (1979) 39, 193

### Short Communication

### THYMECTOMY AND CANCER: A FURTHER REPORT

M. P. VESSEY*, R. DOLL†, B. NORMAN-SMITH* AND I. D. HILL‡

*From the *Department of Social and Community Medicine, Oxford University, the †Department of the Regius Professor of Medicine, Oxford University, and the ‡Division of Computing and Statistics, Northwick Park Hospital, Harrow, Middx.*

Received 6 October 1978 Accepted 8 November 1978

ABOUT 10 years ago, we started an investigation to try to determine whether subjects who had been thymectomized for myasthenia gravis were at an increased risk of extrathymic malignancy. The investigation was prompted by interest in the concept of "immunological surveillance" and by experimental evidence suggesting the likely importance of the thymus in any natural immunological defence mechanisms against cancer. A preliminary report concerned the findings in 383 British patients who underwent thymectomy during the years 1942–64 and who had been successfully followed to the end of 1967 (Vessey & Doll, 1972). Five of these patients had died from extrathymic cancer while 5·5 would have been expected to have done so from the national experience. We report here our findings after an additional 5 years of follow-up.

The total study population consists of 419 patients who underwent thymectomy for myasthenia gravis at the National Hospital for Nervous Diseases, New End Hospital, St Bartholomew's Hospital or the London Hospital between 1 January 1942 and 31 December 1964. The means of identifying the patients is described in detail in our earlier report. Every effort has been made to follow these patients to 31 December 1972 and to obtain death certificates for the deceased. Special enquiries have been made about patients certified as dying from an extrathymic tumour and information has also been sought on the occurrence of non-fatal tumours, by searching case records and writing to family doctors.

Thirty-six of the 419 patients were foreign nationals who came to London specially for thymectomy. These subjects are not considered further, but it should be noted that 34 (94%) have been successfully followed to 31 December 1972. None of these 34 (of whom 11 have died) has, to our knowledge, developed an extrathymic tumour.

Of the remaining 383 patients, all but 2 (both of whom emigrated to the United States) have been followed to 31 December 1972 (a success rate of 99·5%). The characteristics of these patients are given in detail in our earlier report: about two-thirds were female, about one-sixth had a thymoma, and about one half were aged 20–39 at the time of operation.

The number of years that these patients have been at risk of dying have been computed separately for those with and without thymoma, for each sex and 5-year age group, for each 5-year period after thymectomy (0–4, 5–9, *etc.*) and for each 5-year calendar period of observation (1940–44, 1945–49, *etc.*). These numbers have been multiplied by the corresponding death rates for England and Wales, and an estimate has thus been obtained of the numbers of deaths that would be expected if the patients had suffered the same mortality as the general population.

Table I shows the observed and expected numbers of deaths among the 381 patients followed to 31 December, 1972.

TABLE I.—*Observed and expected numbers of deaths by cause according to sex and thymic pathology*

| Sex | Thymic pathology | Total patients | Cause of death: Myasthenia or thymoma Obs | Exp* | Extrathymic tumours Obs | Exp | Respiratory disease (excl. ca) Obs | Exp | Circulatory disease Obs | Exp | Other causes Obs | Exp |
|---|---|---|---|---|---|---|---|---|---|---|---|---|
| M | No thymoma | 85 | 15 | — | 2 | 3·08 | 2 | 1·67 | 9 | 4·65 | 4 | 3·32 |
| M | Thymoma | 23 | 15 | — | 2 | 0·59 | 0 | 0·28 | 1 | 0·82 | 1 | 0·53 |
| F | No thymoma | 231 | 49 | — | 7 | 4·28 | 1 | 0·89 | 3 | 2·84 | 6 | 4·07 |
| F | Thymoma | 42 | 27 | — | 0 | 0·83 | 1 | 0·24 | 1 | 0·86 | 2 | 0·87 |
| | Total | 381 | 106 | — | 11 | 8·78 | 4 | 3·08 | 14 | 9·17 | 13 | 8·79 |

* Expected deaths in this column not computed, but close to zero.

TABLE II.—*Numbers of deaths from extrathymic tumours, observed and expected, by age groups and interval since thymectomy. Patients of both sexes with and without thymoma*

| Age (years) | Interval since thymectomy (years): –4 Obs | Exp | 5–9 Obs | Exp | 10–14 Obs | Exp | 15–19 Obs | Exp | 20+ Obs | Exp | Total Obs | Exp |
|---|---|---|---|---|---|---|---|---|---|---|---|---|
| —19 | 0 | 0·01 | 0 | 0·00 | 0 | 0·00 | 0 | 0·00 | 0 | 0·00 | 0 | 0·01 |
| 20–39 | 0 | 0·20 | 1 | 0·20 | 2 | 0·14 | 0 | 0·07 | 0 | 0·02 | 3 | 0·63 |
| 40–59 | 0 | 0·82 | 0 | 1·02 | 3 | 1·09 | 0 | 1·02 | 0 | 0·92 | 3 | 4·87 |
| 60+ | 0 | 0·23 | 0 | 0·42 | 2 | 0·63 | 0 | 0·92 | 3 | 1·07 | 5 | 3·27 |
| Total | 0 | 1·26 | 1 | 1·64 | 7 | 1·86 | 0 | 2·01 | 3 | 2·01 | 11 | 8·78 |

In addition to the anticipated excess mortality from myasthenia gravis and thymoma, there is a small (and statistically significant) increase in mortality among the thymectomized patients from all other causes, including extrathymic tumours (42 deaths observed, 29·8 expected, $P=0{\cdot}034$). Deaths due to extrathymic tumours alone are slightly, but not statistically significantly, increased (11 observed, 8·8 expected, $P=0{\cdot}5$).

Table II shows how the 11 deaths from extrathymic tumours are distributed with respect to age and interval since thymectomy. The most notable feature is the occurrence of 7 deaths 10–14 years after thymectomy in comparison with only 1·9 expected ($P=0{\cdot}003$). There is, however, no suggestion of an overall relationship between interval since operation and cancer mortality and, while it seems unreasonable to ascribe such an unlikely observation to chance, we have at present no alternative suggestion to offer.

Information concerning the nature of the 11 fatal tumours is given in Table III, which also provides details of 11 non-fatal tumours diagnosed during the follow-up period. These data do not suggest that any particular type of tumour tends to develop after thymectomy. Nor does the number of as yet non-fatal tumours diagnosed suggest that the incidence of cancer (as opposed to the mortality from it) is likely to be raised.

In our earlier report, we concluded that our study provided no evidence that adult thymectomy is followed by an increased risk of neoplastic disease. This conclusion is reinforced by the additional data now available.

Papatestas *et al.* (1977) have presented an analysis of the records of 2000 patients with myasthenia gravis registered at the Mount Sinai Hospital (New York) or at New End Hospital (London). Of these patients, 789 had undergone thymectomy. The risk of developing cancer was calcu-

TABLE III.—*Details of extrathymic tumours.*

| Case no.* | Sex | Thymoma | Age at death | Interval since thymectomy | Nature of tumour |
|---|---|---|---|---|---|
| *Fatal* | | | | | |
| 426 (1) | F | — | 29 | 9 | Hodgkin's disease |
| 154 (2) | F | — | 34 | 13 | Astrocytoma |
| 489 (3) | F | — | 37 | 11 | Osteogenic sarcoma |
| 167 (4) | F | — | 45 | 11 | Ca. breast |
| 357 (5) | F | — | 53 | 14 | Ca. abdominal wall |
| 009 | M | + | 60 | 13 | Ca. stomach |
| 333 | M | + | 69 | 14 | Ca. prostate |
| 152 (7) | M | — | 47 | 13 | Carcinomatosis (1° site unknown) |
| 386 | M | — | 73 | 21 | Pelvic tumour (1° site unknown) |
| 336 | F | — | 62 | 27 | Ca. breast |
| 480 (8) | F | — | 63 | 26 | Chronic lymphatic leukaemia |
| | | | Age at diagnosis | | |
| *Non-fatal* | | | | | |
| 109 (6) | M | + | 43 | 11 | Ca. breast |
| 331 (9) | F | + | 55 | 13 | Bronchial adenoma |
| 104 (10) | F | — | 57 | 16 | Pituitary adenoma |
| 137 | F | + | 67 | 20 | Epithelioma (skin) |
| 443 | F | + | 57 | 13 | Ca. cervix |
| 032 | F | — | 62 | 10 | Ca. breast |
| 142 | F | — | 40 | 10 | Ca. cervix |
| 367 | F | — | 53 | 21 | Ca. breast |
| 404 | F | — | 60 | 26 | Rodent ulcer |
| 433 | F | — | 64 | 15 | Ca. vagina |
| 486 | F | — | 63 | 22 | Ca. recto-sigmoid |

* Numbers in parentheses relate to reference numbers in our 1972 paper.

lated separately in the period of observation after the onset of myasthenia but prior to thymectomy and in the period of observation following thymectomy. Connecticut Cancer Registry data were used to calculate expected numbers of cancers in the two periods. Clearly this approach has a number of major shortcomings (not the least of which is the fact that the patients who underwent thymectomy could not, of necessity, have suffered a fatal cancer prior to the operation) but Papatestas and his colleagues none the less came to the conclusion that myasthenia gravis itself is associated with an increased cancer risk which is reduced by thymectomy. Since all the patients in our study had had a thymectomy, we clearly cannot confirm or refute this suggestion. It should also be noted that there must be some overlap between our data and those of Papatestas *et al.*, but the way in which they present their results prevents any direct comparisons.

Six years ago, the concept of immunological surveillance against neoplasia was almost scientific dogma, but many workers in the field are now expressing doubts about its reality (Möller & Möller, 1976). Our findings, perhaps, add a little weight to the latter view.

We should like to thank the physicians and surgeons at the National Hospital, New End Hospital, St Bartholomew's Hospital and the London Hospital for permission to study patients under their care.

### REFERENCES

MÖLLER, G. & MÖLLER, E. (1976) The concept of immunological surveillance against neoplasia. *Transplant Rev.*, **28**, 2.

PAPATESTAS, A. E., KARK, A. E., GENKINS, G. & AUFSES, A. H. (1977) Protective effect of thymectomy in a high cancer risk population. In *Prevention and Detection of Cancer. Part I. Prevention.* Ed. H. E. Nieburgs. New York: Marcel Dekker. p. 353.

VESSEY, M. P. & DOLL, R. (1972) Thymectomy and cancer—a follow-up study. *Br. J. Cancer*, **26**, 53.